# Supplementary material for: Pyruvate Dehydrogenase Kinase 1 inhibition mediated oxidative phosphorylation enhancement in cartilage promotes osteoarthritis progression
Source: BMC Musculoskelet Disord. 2023 Jul 20;24:597. doi: 10.1186/s12891-023-06585-6 (PMC10357736; doi:10.1186/s12891-023-06585-6)
Supplement: Supplementary file 1 — Additional file 1. [file 12891_2023_6585_MOESM1_ESM.zip › KEGG.Gsea.1653622432464/KEGG_VALINE_LEUCINE_AND_ISOLEUCINE_DEGRADATION.html]

Details for gene set KEGG\_VALINE\_LEUCINE\_AND\_ISOLEUCINE\_DEGRADATION[GSEA]

|  || Dataset | 55457\_collapsed\_to\_symbols.cls.cls #OA\_versus\_normal.cls.cls #OA\_versus\_normal\_repos |
| Phenotype | cls.cls#OA\_versus\_normal\_repos |
| Upregulated in class | OA |
| GeneSet | KEGG\_VALINE\_LEUCINE\_AND\_ISOLEUCINE\_DEGRADATION |
| Enrichment Score (ES) | 0.47188827 |
| Normalized Enrichment Score (NES) | 1.8120446 |
| Nominal p-value | 0.0 |
| FDR q-value | 0.010218428 |
| FWER p-Value | 0.113 |
Table: GSEA Results Summary

  

Fig 1: Enrichment plot: KEGG\_VALINE\_LEUCINE\_AND\_ISOLEUCINE\_DEGRADATION      
 Profile of the Running ES Score & Positions of GeneSet Members on the Rank Ordered List

  

| SYMBOL | TITLE | RANK IN GENE LIST | RANK METRIC SCORE | RUNNING ES | CORE ENRICHMENT || 1 | PCCB | propionyl-CoA carboxylase subunit beta [Source:HGNC Symbol;Acc:HGNC:8654] | 29 | 1.057 | 0.0982 | Yes |
| 2 | DLD | dihydrolipoamide dehydrogenase [Source:HGNC Symbol;Acc:HGNC:2898] | 565 | 0.635 | 0.1170 | Yes |
| 3 | HADHB | hydroxyacyl-CoA dehydrogenase trifunctional multienzyme complex subunit beta [Source:HGNC Symbol;Acc:HGNC:4803] | 675 | 0.599 | 0.1655 | Yes |
| 4 | ALDH2 | aldehyde dehydrogenase 2 family member [Source:HGNC Symbol;Acc:HGNC:404] | 1003 | 0.517 | 0.1892 | Yes |
| 5 | ACAT1 | acetyl-CoA acetyltransferase 1 [Source:HGNC Symbol;Acc:HGNC:93] | 1017 | 0.513 | 0.2370 | Yes |
| 6 | ALDH3A2 | aldehyde dehydrogenase 3 family member A2 [Source:HGNC Symbol;Acc:HGNC:403] | 1263 | 0.465 | 0.2621 | Yes |
| 7 | ACAD8 | acyl-CoA dehydrogenase family member 8 [Source:HGNC Symbol;Acc:HGNC:87] | 1319 | 0.455 | 0.3011 | Yes |
| 8 | HIBCH | 3-hydroxyisobutyryl-CoA hydrolase [Source:HGNC Symbol;Acc:HGNC:4908] | 1526 | 0.424 | 0.3254 | Yes |
| 9 | HMGCL | 3-hydroxy-3-methylglutaryl-CoA lyase [Source:HGNC Symbol;Acc:HGNC:5005] | 1703 | 0.398 | 0.3496 | Yes |
| 10 | HMGCS2 | 3-hydroxy-3-methylglutaryl-CoA synthase 2 [Source:HGNC Symbol;Acc:HGNC:5008] | 1888 | 0.374 | 0.3708 | Yes |
| 11 | ECHS1 | "enoyl-CoA hydratase, short chain 1 [Source:HGNC Symbol;Acc:HGNC:3151]" | 1925 | 0.369 | 0.4032 | Yes |
| 12 | ALDH7A1 | aldehyde dehydrogenase 7 family member A1 [Source:HGNC Symbol;Acc:HGNC:877] | 2158 | 0.343 | 0.4178 | Yes |
| 13 | BCKDHB | branched chain keto acid dehydrogenase E1 subunit beta [Source:HGNC Symbol;Acc:HGNC:987] | 2765 | 0.273 | 0.3966 | Yes |
| 14 | ACAA2 | acetyl-CoA acyltransferase 2 [Source:HGNC Symbol;Acc:HGNC:83] | 2779 | 0.272 | 0.4215 | Yes |
| 15 | ALDH9A1 | aldehyde dehydrogenase 9 family member A1 [Source:HGNC Symbol;Acc:HGNC:412] | 2795 | 0.270 | 0.4460 | Yes |
| 16 | OXCT1 | 3-oxoacid CoA-transferase 1 [Source:HGNC Symbol;Acc:HGNC:8527] | 2897 | 0.261 | 0.4630 | Yes |
| 17 | MMUT | methylmalonyl-CoA mutase [Source:HGNC Symbol;Acc:HGNC:7526] | 3362 | 0.215 | 0.4474 | Yes |
| 18 | ACADSB | acyl-CoA dehydrogenase short/branched chain [Source:HGNC Symbol;Acc:HGNC:91] | 3382 | 0.213 | 0.4662 | Yes |
| 19 | BCAT2 | branched chain amino acid transaminase 2 [Source:HGNC Symbol;Acc:HGNC:977] | 3552 | 0.198 | 0.4719 | Yes |
| 20 | OXCT2 | 3-oxoacid CoA-transferase 2 [Source:HGNC Symbol;Acc:HGNC:18606] | 4035 | 0.152 | 0.4489 | No |
| 21 | HSD17B10 | hydroxysteroid 17-beta dehydrogenase 10 [Source:HGNC Symbol;Acc:HGNC:4800] | 4060 | 0.150 | 0.4613 | No |
| 22 | HADHA | hydroxyacyl-CoA dehydrogenase trifunctional multienzyme complex subunit alpha [Source:HGNC Symbol;Acc:HGNC:4801] | 4163 | 0.141 | 0.4668 | No |
| 23 | MCCC2 | methylcrotonyl-CoA carboxylase subunit 2 [Source:HGNC Symbol;Acc:HGNC:6937] | 4363 | 0.123 | 0.4631 | No |
| 24 | IVD | isovaleryl-CoA dehydrogenase [Source:HGNC Symbol;Acc:HGNC:6186] | 4950 | 0.076 | 0.4247 | No |
| 25 | ACADM | acyl-CoA dehydrogenase medium chain [Source:HGNC Symbol;Acc:HGNC:89] | 4983 | 0.074 | 0.4293 | No |
| 26 | HADH | hydroxyacyl-CoA dehydrogenase [Source:HGNC Symbol;Acc:HGNC:4799] | 5088 | 0.065 | 0.4274 | No |
| 27 | ACAA1 | acetyl-CoA acyltransferase 1 [Source:HGNC Symbol;Acc:HGNC:82] | 5175 | 0.058 | 0.4262 | No |
| 28 | ALDH6A1 | aldehyde dehydrogenase 6 family member A1 [Source:HGNC Symbol;Acc:HGNC:7179] | 5560 | 0.028 | 0.3990 | No |
| 29 | BCKDHA | branched chain keto acid dehydrogenase E1 subunit alpha [Source:HGNC Symbol;Acc:HGNC:986] | 5671 | 0.018 | 0.3921 | No |
| 30 | AUH | AU RNA binding methylglutaconyl-CoA hydratase [Source:HGNC Symbol;Acc:HGNC:890] | 5911 | 0.001 | 0.3737 | No |
| 31 | AOX1 | aldehyde oxidase 1 [Source:HGNC Symbol;Acc:HGNC:553] | 6243 | -0.024 | 0.3502 | No |
| 32 | ACAT2 | acetyl-CoA acetyltransferase 2 [Source:HGNC Symbol;Acc:HGNC:94] | 6291 | -0.027 | 0.3491 | No |
| 33 | ALDH1B1 | aldehyde dehydrogenase 1 family member B1 [Source:HGNC Symbol;Acc:HGNC:407] | 6551 | -0.046 | 0.3333 | No |
| 34 | PCCA | propionyl-CoA carboxylase subunit alpha [Source:HGNC Symbol;Acc:HGNC:8653] | 6627 | -0.052 | 0.3324 | No |
| 35 | ABAT | 4-aminobutyrate aminotransferase [Source:HGNC Symbol;Acc:HGNC:23] | 6804 | -0.063 | 0.3247 | No |
| 36 | BCAT1 | branched chain amino acid transaminase 1 [Source:HGNC Symbol;Acc:HGNC:976] | 7106 | -0.088 | 0.3097 | No |
| 37 | MCCC1 | methylcrotonyl-CoA carboxylase subunit 1 [Source:HGNC Symbol;Acc:HGNC:6936] | 7401 | -0.111 | 0.2974 | No |
| 38 | EHHADH | enoyl-CoA hydratase and 3-hydroxyacyl CoA dehydrogenase [Source:HGNC Symbol;Acc:HGNC:3247] | 7968 | -0.153 | 0.2679 | No |
| 39 | ACADS | acyl-CoA dehydrogenase short chain [Source:HGNC Symbol;Acc:HGNC:90] | 9105 | -0.240 | 0.2023 | No |
| 40 | HMGCS1 | 3-hydroxy-3-methylglutaryl-CoA synthase 1 [Source:HGNC Symbol;Acc:HGNC:5007] | 10829 | -0.408 | 0.1071 | No |
| 41 | DBT | dihydrolipoamide branched chain transacylase E2 [Source:HGNC Symbol;Acc:HGNC:2698] | 11873 | -0.566 | 0.0799 | No |
Table: GSEA details [plain text format]

  

Fig 2: KEGG\_VALINE\_LEUCINE\_AND\_ISOLEUCINE\_DEGRADATION      
 Blue-Pink O' Gram in the Space of the Analyzed GeneSet

  

Fig 3: KEGG\_VALINE\_LEUCINE\_AND\_ISOLEUCINE\_DEGRADATION: Random ES distribution      
 Gene set null distribution of ES for **KEGG\_VALINE\_LEUCINE\_AND\_ISOLEUCINE\_DEGRADATION**
